# Correlation of Serum Anti-Mullerian Hormone with Ovarian Follicle Output Rate in Infertile Females: A Clomiphene Citrate Challenge Test

**DOI:** 10.7759/cureus.8032

**Published:** 2020-05-08

**Authors:** Syeda Fatima, Qanbar Naqvi, Ambreen Tauseef, Mehwish Qamar, Qudsia U Khan, Tanzeela Akram, Muniza Saeed

**Affiliations:** 1 Physiology, Islam Medical and Dental College, Sialkot, PAK; 2 Anatomy, Islam Medical and Dental College, Sialkot, PAK; 3 Physiology, Combined Military Hospital Lahore Medical College and Institute of Dentistry, Lahore, PAK; 4 Physiology, Ameer Ud Din Medical College, Lahore, PAK

**Keywords:** infertility, anti-mullerian hormone, fort, clomiphene citrate challenge test

## Abstract

Background

Failure to achieve a successful pregnancy after 12 months of unprotected intercourse is a pathology of the reproductive system known as infertility. Anti-Mullerian hormone (AMH) not only reflects the ovarian reserve but also is known to be a predictor of several assisted reproductive techniques, e.g., in vitro fertilization (IVF), intracytoplasmic sperm injection (ICSI), and clomiphene citrate challenge test. In this study, AMH levels are correlated with the follicular output rate after the clomiphene citrate challenge test.

Objective

The objective of this study is to correlate AMH with the follicular output rate (FORT) after the clomiphene citrate challenge test.

Materials and methods

This study included a total of 80 primary and secondary infertile females, divided into early (18-30) and late (31-45) reproductive age groups either currently under clomiphene citrate treatment or advised to start clomiphene treatment, culled from out-patient department and centers of assisted reproductive techniques. On the third day of the menstrual cycle, blood samples were taken to determine serum AMH levels by ELISA. Then on the fifth day of the menstrual cycle, antral follicular counts were calculated through transvaginal ultrasound and oral tablet clomiphene citrate was started, and on day 12 and then on day 5, transvaginal ultrasound was repeated to record the number and diameter of dominant follicles.

Results

The pre-ovulatory (mature) follicle count was divided by the antral follicle count ×100 for calculating the FORT, which showed a negative Spearman Rho correlation (*p *= 0.048) with AMH. P-value ≤ 0.05 was considered significant.

Conclusion

It is concluded that the most commonly administered drug, clomiphene citrate, may not be the treatment of choice for patients with high levels of AMH and may, in fact, interfere with the chances of achieving pregnancy. This study can provide guidelines to clinicians for patient counseling, given the results of the clomiphene citrate challenge test on the basis of AMH levels.

## Introduction

Infertility is characterized as the inability of a couple to conceive from unprotected intercourse after an entire year or, in the case of women above 35 years of age, after six cycles of unprotected intercourse [1]. According to the World Health Organization, 50 to 80 million couples suffer from infertility, of whom 30% are diagnosed as idiopathic infertile couples [1-2].

There is a well-established parallel link between increasing maternal age and decline of fertility for both natural ovarian cycles and in patients treated with controlled ovarian hyper-stimulation, starting in the second and trailing off in the third decade of life [3]. This waning of fertility with age is primarily caused and reflected by the decrease in “ovarian reserve” that defines the quantity as well as the quality of ovarian follicular pool [4].

Several ovarian reserve tests have been developed over the past decades that give an approximate and indirect estimate of a female’s remaining fertile years [5]. Of all the available ovarian reserve markers, anti-Mullerian hormone (AMH) has emerged as the most promising one because it reflects the number of growing ovarian follicles [6]. The granulosa cells of these growing antral follicles secrete measurable serum levels of AMH, making it a reliable marker for ovarian aging [7]. AMH levels also allow the treatment of infertility to be tailored to each individual [8].

Follicular output rate (FORT), calculated as pre-ovulatory count ratio to antral follicle count (AFC)x 100, has been proposed as a valuable gauge for estimating follicular response to controlled ovarian hyperstimulation [9]. Among various tests used for assessing ovarian response, the clomiphene citrate challenge test is the best-documented and most commonly used [10]. Hence, this study aims to determine the correlation of serum anti-mullerian hormone and follicle output rate after the clomiphene citrate challenge test.

## Materials and methods

This correlational study was conducted in the Assisted Conception Unit and Obstetrics and Gynecology units of two tertiary care hospitals after obtaining approval from the ethical committee of Post Graduate Medical Institute Lahore. 

The sample size was estimated using the WHO formula, with a confidence interval of 95%. Eighty (80) females with idiopathic primary or secondary infertility were selected on day 3 of their menstrual cycle, using non-probability purposive sampling. After obtaining informed consent, social-demographic details were recorded. The patient’s height and weight were noted and BMI was calculated. On the third day of each subject’s menstrual cycle, five milliliters of blood sample were drawn, centrifuged for 15 minutes (at 3,000 rpm), and labeled. The aliquoted serum was stored at -20°C within 24 hours of collection. Serum levels of AMH were measured via the using enzyme-linked immunosorbent (ELISA) assay test kit from Bio-Check, Inc. USA. On day 5 of the menstrual cycle, AFC was performed via transvaginal ultrasound and 100 mg oral clomiphene citrate was administered from day 5 to day 9 of the same menstrual cycle [10].

Transvaginal ultrasound was repeated on the 12th day of the same cycle, and the number and diameter of mature follicles were recorded. A group of follicles in the size range of 18-28 mm was designated dominant or mature ovarian follicles [11]. FORT was calculated using the antral follicle count and pre-ovulatory follicle count.

Statistical analysis

The data were entered and analyzed using Social Package of Statistical Sciences (SPSS) version 21. The normality of the data was assessed using the Kolmogorov-Smirnov test. The quantitative variables were presented as median (IQR), and categorical data were presented as frequency and percentage.

Spearman's Rho correlation was applied to find out the correlation between AMH and FORT. A p-value ≤ 0.05 was considered significant.

## Results

In this study, serum AMH was stratified into five groups: very low (<0.3 ng/ml), low (0.3-0.6 ng/ml), low normal (0.7-0.9 ng/ml), normal (1-3 ng/ml), and high (>3 ng/ml) levels. The majority of the patients in the late reproductive age group presented with AMH levels in the low range, but 66.7% of the patients in the early reproductive age group presented with high AMH levels, demonstrating a highly significant difference between the two age groups (p<0.001). Follicular output rate measurement showed no significant difference between the two age groups (Table 1).

**Table 1:** Frequency distribution and comparison of serum AMH between early and late reproductive age groups AMH, anti-Mullerian hormone; IQR: Interquartile range; **Comparison by Mann-Whitney U test; *Difference is significant ≤0.05 level

Variables		Early reproductive age group (18-30) n=60 Frequency (%)	Late reproductive age group(31-40) n=22 Frequency (%)	p-value
AMH (ng/ml)	Very low (<0.3)	12(20%)	8(36.4%)	0.000*
	Low (0.3-0.6)	2(3.3%)	2(9.1%)	
	Low normal (0.7-0.9)	1(1.7%)	3(13.6%)	
	Normal (1.0-3.0)	5(8.3%)	4(18.2%)	
	High (>3)	40(66.7%)	5(22.7%)	
**Median (IQR)		4.2(2.6-5.4)	1.4(0.37-3.05)	

A significant negative correlation (0.048) was observed between AMH and FORT, indicating a decrease in the follicular output rate with an increase in AMH (Table 2).

**Table 2:** Correlation of AMH with age and FORT using Spearman's Rho correlation test **Correlation is significant ≤0.01 level (2-tailed); *Correlation is significant ≤0.05 level (2-tailed); AMH: anti-Mullerian hormone; FORT: follicular output rate

Variables	Rho	P-value
Age (years)	-0.365	0.001**
FORT	-0.219	0.048*

## Discussion

This study measured the difference in the levels of AMH in different age groups, and the correlation between AMH and FORT revealed a significant difference in AMH levels between different age groups along with a significant negative correlation between FORT and AMH. The patient population selected for the study was stratified into 5 groups of AMH [12]. The majority of the early age group had AMH levels in high range, and the majority of the late group had AMH levels in the low range, indicating a significant (p<0.001) decline in the levels of AMH with advancing age. A similar significant difference of AMH in two reproductive age groups was also found by Hiremath et al. and Meczekalski et al., who reported similar results by studying the role of AMH in healthy women of late reproductive age and recommended AMH as the best endocrine marker for assessing age-related decline in fertility [12-13]. However, Andersen et al. measured the concentration of AMH and inhibin B in follicular fluid from small antral follicles and observed an insignificant difference in AMH values among age groups [14]. This difference might be attributed to the gonadotoxic treatment administered for malignant disease. FORT has been employed in the past as a tool for assessing ovarian response to stimulation by gonadotropins or other modalities [15]. In the present study, clomiphene citrate was used as an ovarian stimulant based on its mechanism of desensitizing or blocking the effect of estrogen on the hypothalamic-pituitary axis, thereby increasing FSH output, leading to follicular recruitment and maturation [16]. On the other hand, AMH showed an effect of decreasing initial follicular enrolment and inhibiting FSH during the growth and selection of follicles [17]. A significant negative correlation was found between AMH and FORT (p=0.048), consistent with the study by Genro et al., who were first to introduce the concept of FORT and who concluded that high levels of AMH inhibit the sensitivity of follicles to FSH and thus lower the follicular output rate [18-19]. Grynberg et al. supported these results by reporting a significant decrease in FORT from low to high AMH levels [15]. Bessow et al. indirectly supported similar results, by reporting no correlation between FORT and AFC >6 and establishing higher levels of AMH as the cause of decreasing follicular responsiveness to FSH in patients with higher AFC [20]. 

## Conclusions

Clomiphene citrate is treated as an over-the-counter drug, prescribed without much insight into the psychological impact it has on the infertile couples. Therefore, AMH testing, if established as a standard of care, can prevent such rampant over-prescription of this drug because high levels of AMH decrease or inhibit the effect of clomiphene citrate on follicles and thereby decrease FORT and a chance of achieving pregnancy.
